# Nicotine delivery and users’ reactions to Juul compared with cigarettes and other e‐cigarette products

**DOI:** 10.1111/add.14936

**Published:** 2020-01-29

**Authors:** Peter Hajek, Kate Pittaccio, Francesca Pesola, Katie Myers Smith, Anna Phillips‐Waller, Dunja Przulj

**Affiliations:** ^1^ Health and Lifestyle Research Unit Queen Mary University of London London UK; ^2^ Faculty of Life Sciences and Medicine King's College London London UK

**Keywords:** E‐cigarettes, Juul, nicotine, nicotine delivery, pharmacokinetic, tobacco

## Abstract

**Aims:**

To assess the pharmacokinetic (PK) profile of, and users’ reactions to, Juul (59 mg nicotine/ml) as an indication of its therapeutic and dependence potential.

**Design:**

Cross‐over, within‐subjects study in which participants attended after overnight abstinence on separate sessions and smoked a cigarette or used Juul or eight other types of e‐cigarettes (EC) *ad libitum* for 5 minutes. The Juul product used was the version available in the United States that has more nicotine in the e‐liquid than the one available in the European Union.

**Setting:**

Laboratory setting in the United Kingdom.

**Participants:**

Twenty dual users (smokers who also vape) provided data on Juul and cigarettes, with eight also providing data on other EC products.

**Measurements:**

At each session, number of puffs taken was counted during the 5‐minute product use period and blood samples were taken at baseline and at 2, 4, 6, 8, 10 and 30 minutes after starting smoking/vaping and analysed for nicotine. Participants also monitored their urges to smoke and rated the products on a range of characteristics.

**Findings:**

Juul's PK profile was close to the PK profile of cigarettes [maximum concentration (C_max_) = 20.4 versus 19.2 ng/ml; time to maximum concentration (T_max_) = 4 versus 6 minutes; area under the curve (AUC): 307.9 versus 312.6, respectively]. Compared with other EC products, Juul had shorter T_max_ [4 minutes, (IQR = 2.5–4.0) versus 6.3 minutes, (IQR = 4.7 – 8.1), *P* = 0.012] and higher C_max_ (28.9 (SD = 15.6) versus 10.6 (SD = 5.5), *P* = 0.013) despite a lower number of puffs (12.5 (SD = 4.2) versus 17.0 (SD = 4.2), *P* = 0.084). Compared with other e‐cigarette products, it also provided faster reduction of urges to smoke and obtained more favourable subjective ratings.

**Conclusion:**

Juul's PK profile and user ratings suggest that it could be more effective than other EC products in helping smokers to quit smoking, but it may also have a higher potential to generate regular use in non‐smokers.

## Introduction

Juul is a novel cartridge‐based electronic cigarette (EC) that is currently at the centre of intense interest and controversy. It has been outselling other types of EC in the United States 1, 2, and it seems to be increasingly popular both among established smokers who want to quit smoking but also among young people 3, 4. This raises a concern that adolescents who have not smoked previously may not only be experimenting with the device but also becoming regular Juul users, although data on whether and to what extent this is happening were not available at the time of writing.

Juul's commercial success could be due to several of its innovative features. It can be charged directly from a USB port and does not require filling up or cleaning, which makes it much easier to use than refillable ECs that previously dominated this market 5. More importantly, however, it uses nicotine salt rather than freebase nicotine. This has more physiological pH, which avoids alkaline activation of protective mechanisms located in the airways and makes inhalation less harsh 6. This could be the mechanism that makes it possible to include 59 mg/ml nicotine in Juul's e‐liquid, a level much higher than 18 mg/ml, which is the most popular nicotine concentration among regular users of refillable EC 7. There is no expectation that Juul provides three times higher nicotine delivery than its competitors, as there is only a relatively narrow range of nicotine concentration from acute use that smokers find rewarding 8. However, even advanced ECs deliver less nicotine than cigarettes and do it more slowly 9. Juul may be providing nicotine in a way that is closer to the pharmacokinetic (PK) profile of nicotine delivery from cigarettes. 1Note that the nicotine solution in the version of Juul that is available in the European Union has to comply with the EU limit of maximum of 20 mg/ml. This study concerns the full‐strength US version.


Alternative nicotine delivery devices that have PK characteristics closer to smoking are likely to be more effective in helping smokers quit. If Juul indeed has faster and higher nicotine delivery than other EC products, it would signal better treatment potential. A PK profile closer to smoking can be expected to also increase the likelihood of the product's long‐term use. Among nicotine replacement treatments (NRT), the highest rate of long‐term use has been noted with nicotine nasal spray that provides the fastest nicotine delivery, followed by oral products that are slower, followed by nicotine patches that are the slowest 10, 11. Ongoing use of less harmful nicotine products in smokers who cannot or do not want to stop nicotine use altogether may generate some adverse health effects over the long term and maintain nicotine dependence, but it may also have a positive impact if it prevents withdrawal discomfort and weight gain and/or protects such users from relapse to smoking 12. However, the speed of nicotine delivery that is close to that of cigarettes could also increase the likelihood that non‐smokers who try the product become regular users. Existing ECs can satisfy smokers and help them to stop smoking 13, but they seem to be remarkably unattractive to non‐smokers. While non‐smokers who try cigarettes have a more than 50% chance of becoming daily smokers 14, non‐smokers who try ECs almost never progress to weekly, let alone daily, use 15, 16. There is now a concern that use of Juul may be changing this picture 17.

Limited data exist on Juul's PK profile and user reactions to the product that would allow an assessment of its therapeutic and dependence potential. We are aware of only one conference poster by the manufacturers that suggests only a slightly lower and slower nicotine intake from Juul compared to cigarettes, but the methodology of this study is unclear (only one product was used after overnight abstinence, and it is not clear which one), participants were not allowed to use their own cigarette, and no comparison with other EC products was included 18.

We conducted the first independent study, to our knowledge, of Juul's PK profile and user reactions. The purpose of the study was to compare nicotine delivery from Juul with nicotine delivery from cigarettes and from other types of EC. We also compared these products in their effects on urges to smoke after overnight abstinence and in user ratings. If Juul provides better nicotine delivery and withdrawal relief than other ECs and closer to what cigarettes provide, this would have implications for both its therapeutic and dependence potential.

## Methods

### Aim and design

This was a cross‐over within‐subjects study to establish the PK profile of Juul and to compare it with the PK profile obtained from participants’ own cigarettes and from other EC products; and to assess user reactions across the products.

### Participants

Twenty healthy adults who were currently vaping daily and also smoking at least occasionally (‘dual users’) and who were willing to test a series of EC products were recruited via social media and by word of mouth. Dual users were recruited, as PK data from smoking were required and it would not be ethical to ask ex‐smokers to smoke.

### Procedures

All participants provided informed consent. Participants attended the laboratory after overnight abstinence from both smoking and vaping. Their abstinence from smoking for at least 10 hours was confirmed by a carbon monoxide (CO) reading of < 10 parts per million (p.p.m.).

At the first session, they smoked a cigarette of their usual brand which they brought with them. At the next session, Juul was tested. Sessions were scheduled with at least a 1‐week ‘wash‐out’ period between them.

Eight of the 20 participants also took part in a previous study 9, 19 where eight different EC products were tested in exactly the same manner (see below). 2Note that all products, including Juul and cigarettes, were tested in the same order, rather than in a random order. In this ongoing project, where new products are tested when they appear, order of testing cannot be randomized, but the standard order ensures that all participants had the same prior experience and frame of reference when rating the products and comparing them to each other. The sessions started between 7.30 and 9.30 a.m., depending on participants’ availability, and took approximately 60 minutes.


At each session, an intravenous line for blood sampling was placed in the forearm and a baseline blood sample (up to 5 ml) was taken, after which participants were asked to smoke/vape *ad libitum* for 5 minutes (‘You can now use your cigarette/e‐cigarette as much or as little as you want for 5 minutes’). Further blood samples were taken at 2, 4, 6, 8, 10 and 30 minutes after starting smoking/vaping.

Blood was stored at −20°C and couriered to the laboratory for analysis within 7 days.

Participants received £60 at the end of each session.

## Measures

Demographic and smoking history data were collected at baseline. The number of puffs taken was counted during the 5‐minute product use period. Urges to smoke were rated at baseline and at 5, 10, 15 and 30 minutes, on a scale of 1 (‘no urge at all’) to 10 (‘extreme urge’). The measure was used in our previous reports 9, 19.

At the end of each session, products were evaluated as in our previous report 19. The following questions were asked at both the cigarette and EC/JUUL sessions (rated on a scale of 1–10): ‘Did it relieve your urge to smoke?’ [not at all (1), extremely well (10)]; ‘How quickly did any effect happen?’ [very slowly (1), extremely fast (10)]; ‘How much nicotine do you think it delivered?’ [too little (1), just right (5), too much (10)]; ‘Did you like the taste?’ [not at all (1), extremely (10)]; ‘Was it pleasant to use?’ [not at all (1), extremely (10)].

The following additional questions were asked at the EC/JUUL sessions: ‘How comfortable was the mouthpiece?’ [not at all (1), extremely (10)]; ‘How would you rate the amount of vapour the product produced?’ [too little (1), just right (5), too much (10)]; ‘How likely would you be to recommend it to friends?’ [not at all (1), extremely (10)]; ‘Overall, how does it compare with your usual e‐cigarette?’ [much worse (1), about the same (5), much better (10)]. Two other questions, ‘How hard was it to draw smoke/vapour from it?’ [too easy (1), just right (5), too hard (10)] and ‘How would you rate the ‘hit’/‘scratch’ at the back of your throat it provided?’ [too little (1), just right (5), too much (10)] were included at a later date, so only 12 of the 20 participants completed them.

Blood samples were analysed for nicotine at ABS Laboratories Ltd, BioPark (Welwyn Garden City, UK), using capillary column gas chromatography with detection by electron impact mass spectrometry and selected ion monitoring 20. PK parameters included maximum nicotine concentration (C_max_), time to the maximum (T_max_) and area under the curve [AUC_0 ≥ 30_], a measure of total nicotine delivery over 30 minutes 21. All measures were corrected for baseline values.

### Study products

We tested Juul, 59 mg/ml nicotine content, with Virginia Tobacco flavour (JUUL Labs), and participants supplied their own‐brand cigarettes. This form of the product is available in the United States but not in the European Union, where the maximum nicotine concentration allowed is 20 mg/ml.

For eight participants, data were also available on eight other EC brands, all tested with tobacco‐flavoured e‐liquids and with nicotine concentrations as close to 20 mg/ml as possible, plus one product with the highest nicotine content available at the time (Vuse, 48 mg/ml nicotine). Six were first‐generation cig‐a‐like products: Gamucci (16 mg/ml nicotine), Blu (18 mg/ml), Vype (16.8 mg/ml), E‐lites (24 mg/ml), Puritane (20 mg/ml) and Vuse (48 mg/ml). Two were refillable products: KangerTech EVOD and variable voltage product Innokin iTaste MVP 2, set to 4.8 V (range = 3.3–5.0 V). Both of these were used with the same 20 mg/ml nicotine e‐liquid (for details of this earlier study, see 9, 19).

### Statistical analysis

PK parameters were calculated using PKSolver add‐in for Excel version 2.0 22. The following PK parameters were calculated: (1) time at which the highest nicotine concentration occurred in plasma (T_max_); (2) the highest drug concentration observed in plasma (C_max_); (3) estimated area under the plasma nicotine curve concentration from time 0 to 30 minutes (AUC_0 ≥ 30_). Nicotine concentrations in post‐use blood samples were corrected for baseline levels. C_max_, T_max_ and AUC_0 ≥ 30_ were estimated using a non‐compartmental model and trapezoidal rule. Non‐compartmental analysis (NCA) is a frequently used method in PK analysis (for the theory and details of NCA calculation see 23).

Differences between products were analysed using a paired‐sample *t*‐test or a non‐parametric equivalent when parametric assumptions were not met. We examined the main effects of time and product as well the time × product interaction on urges to smoke rated over the 30‐minute testing period using a mixed effect model with robust standard errors. We used patients as the cluster and random slopes and intercepts to account for repeated measures. Analyses were adjusted for baseline urge scores recorded at time 0 (before any product use). The Wald test was used to assess the overall significance of time and the time × product interaction.

The normality assumption was assessed using the Shapiro–Wilks test and through visual inspection using normal probability plots to assess the normality of the difference score between the two dependent variables for paired *t*‐tests and (b) standardized residuals for the mixed model. Moreover, for the mixed model we visually assessed the assumption of homoscedasticity by plotting the standardized residuals against the fitted values. Analyses were performed with SPSS version 25, except for the mixed models which were performed with Stata version 15.

The study was not pre‐registered on a publicly available platform, so the results should be considered exploratory.

The project was approved by the QMUL Ethics of Research Committee on 3 April 2018 (QMERC2018/09).

## Results

Table 1 shows sample characteristics. The median age of the sample was 30 years; their mean cigarette consumption prior to starting EC use was 15 cigarettes per day, while at the time of joining the study this was just over one cigarette per day (range = 1–50 cigarettes per week). Cigarette brands used are shown in Supporting information, Table S1. Four participants were using menthol cigarettes. The high nicotine content Juul is not available in the United Kingdom and no participant had had prior experience with it, but two participants had experience with the UK version of Juul (18 mg/ml nicotine). A total of 19 (95%) participants used refillable ECs. Mean nicotine concentration in participants’ own e‐liquids was 12 ng/ml [standard deviation (SD) = 6]. Among the 11 different products we tested, baseline nicotine levels were low, but in 5% of the sample (*n* = 6) individual participants had baseline nicotine levels of > 10 ng/ml (11.3–21.5 ng/ml), suggesting nicotine intake late at night or in the morning.

**Table 1:** Participant characteristics.

Age, median (IQR)	30 (27–41)
Male *n* (%)	16 (80)
Higher education *n* (%)	12 (60)
Cigarettes smoked per day before starting EC use, median (IQR)	15 (8–20)
Fagerstrom Test for Cigarette Dependence (FTCD) before EC use, median (IQR)	4 (1.5–6)
Cigarettes smoked per day when joining the study, median (IQR) (*n* = 19)	0.7 (0.6–2.0)
EC cartridges used per day, (*n* = 1)	2
Millilitres of e‐liquid used per day, median (IQR) (*n* = 19)	2 (1–4.3)
No. months using EC daily, median (IQR)	12 (3–36)
Days EC used in last week, median (IQR)	7 (7–7)

### Comparisons of Juul and cigarettes

**Figure 1:**
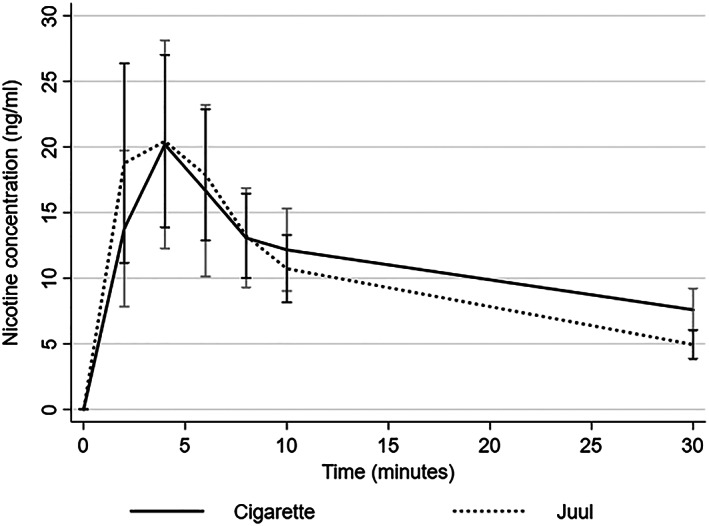
Pharmacokinetic (PK) profiles of own brand cigarette and Juul (mean scores with 95% confidence interval)

PK profiles achieved with Juul and with own‐brand cigarettes are shown in Fig. 1.

Table 2 shows puffs taken and PK parameters for each product. The PK profile of the two products and the puffing rates are shown in Table 2. Juul had a faster T_max_, but the difference did not reach statistical significance (*z* = −2.0, *P* = 0.051).

**Table 2:** Nicotine delivery and number of puffs taken from own‐brand cigarette and from Juul.

Product	Mean no. of puffs (SD)	Mean C_max_ (SD)	Median T_max_ a (IQR)	Mean AUC_0 ≥ 30_ a (SD)
Cigarette	13.3 (3.8), range = 5–19	19.2 (17.6)	6 (4–8)	312.6 (187.6)
Juul	15.3 (6.0), range = 8–28	20.4 (15.0)	4 (2–6)	307.9 (172.5)
Difference^b^	*t*_(19)_ = −1.5, *P* = 0.15	*t*_(19)_ = −0.3, *P* = 0.76	*z* = −2.0, *P* = 0.051	*t*_(19)_ = 0.1, *P* = 0.91
Effect size^c^	*d* = 0.34	*d* = 0.07	*R* = 0.45	*d* = 0.03

a Median time to maximum concentration (T_max_) values and mean area under the curve (AUC) values that were used to compare electronic cigarette (EC) products statistically differ slightly from values in Fig. 1 estimated by PKSolver, because the comparisons used means across individuals whereas PKSolver calculates means across time‐points.

b Paired *t*‐test was used if parametric assumptions were met and Wilcoxon's signed‐ranked test if not.

c Effect size was estimated using Cohen's *d*‐test following paired *t*‐test – *t*/sqrt(*n*) or following a Wilcoxon's signed‐rank test – *z*/sqrt(*n*). SD = standard deviation; C_max_ = maximum concentration.

**Figure 2:**
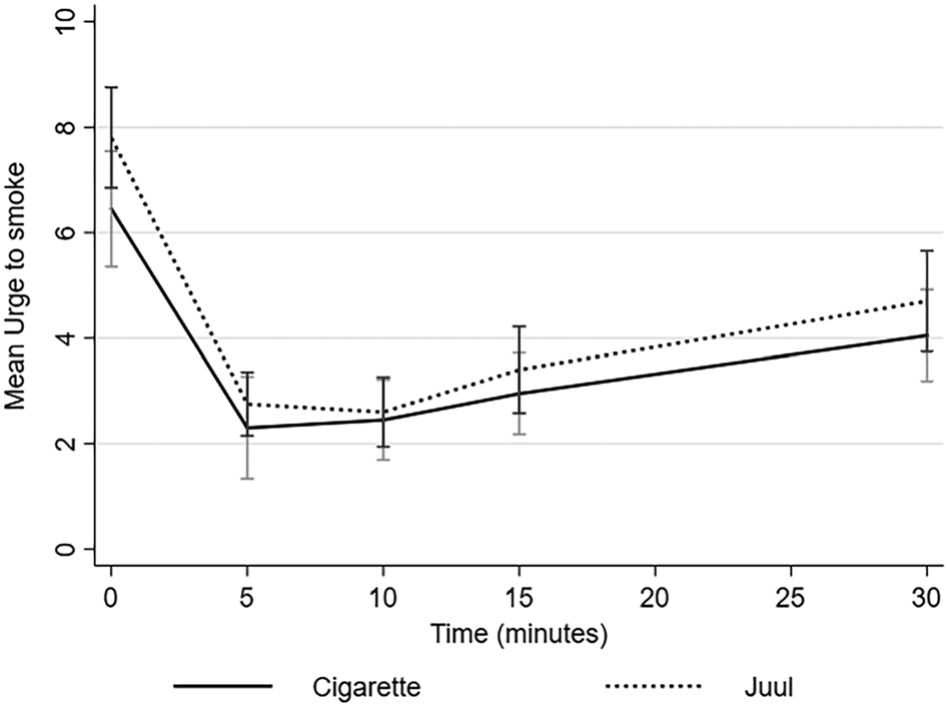
Effects of cigarettes and Juul on urges to smoke (mean scores with 95% confidence interval)

Concerning urges to smoke, the mixed model regression showed a main effect of time (Wald χ^2^_(3)_ = 21.9, *P* < 0.001). Figure 2 shows that urges to smoke subsided between baseline and 5 minutes and subsequently increased without reaching the levels observed at baseline. There was no main effect of product (Wald χ^2^_(1)_ = 0.1, *P* = 0.73) and no significant interaction between product and time (Wald χ^2^_(3)_ = 2.8, *P* = 0.42) (see Supporting information, Table S2).

Table 3 compares Juul and cigarettes in other ratings. Juul matched cigarettes closely across different items.

**Table 3:** Ratings of Juul and own cigarette.

Product characteristic	Juul^c^	Own cigarette^c^	Difference^a^	Effect size^b^
Did it relieve your urge to smoke? (1–10)	9.0 (8.0–10.0)	10.0 (8.–10.0)	*z* = −0.1 *P* = 0.91	0.002
How quickly did any effect happen? (1–10)	7.9 (1.7)	7.0 (2.0)	*t*_(19)_ = −1.8 *P* = 0.08	0.40
Subjective nicotine delivery (1 = too little, 5 = just right, 10 = too much)	6.2 (1.8)	5.4 (1.8)	*t*_(19)_ = −1.5 *P* = 0.15	0.34
Taste	5.9 (2.5)	5.4 (2.7)	*t*_(19)_ = −0.74, *P* = 0.47	0.17
Pleasantness	7.0 (6.0–9.8)	10.0 (8.0–10.0)	*z* = −0.94 *P* = 0.35	0.21

a Paired *t*‐test if parametric assumptions were met; Wilcoxon's sign‐ranked test if not.

b Effect size was estimated using Cohen's *d*‐test following paired *t*‐test – *t*/sqrt(*n*) or *r* following Wilcoxon's signed‐rank test – *z*/sqrt(*n*).

c Mean (SD) and *t*‐test are reported if assumptions are met; median (IQR) and Wilcoxon test reported if not.

### Comparisons of Juul and other EC products

**Figure 3:**
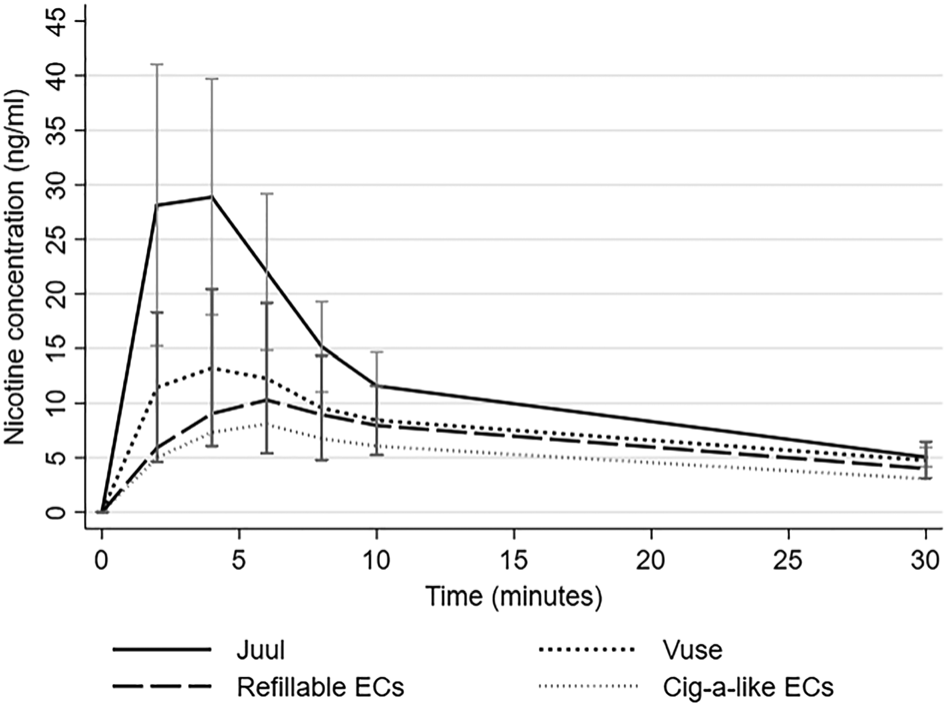
Pharmacokinetic (PK) profiles of eight conventional electronic cigarette (EC) products and Juul (mean scores with 95% confidence intervals)

Figure 3 compares PK profiles of Juul and the traditional EC products in a sample of eight participants who provided complete data. The five cig‐a‐like and two refillable products had similar PK characteristics, so their scores were averaged. One of the cartridge‐based products, Vuse, included e‐liquid with 48 mg/ml nicotine (compared to approximately 20 mg/ml in all other products), and is shown separately.

Juul had significantly shorter T_max_ and higher C_max_ and AUC than other EC products [T_max_ 4 minutes (IQR = 2.5–4.0) versus 6.3 minutes (IQR = 4.7 – 8.1), *z* = −2.5, *P* = 0.012; C_max_ = 28.9 (SD = 15.6) versus 10.6 (SD = 5.5), *t*_(7)_ = − 3.3, *P* = 0.013; AUC = 366.4 (SD = 153.9) versus 200.5 (SD = 78.8), *t*_(7)_= − 3.0, *P* = 0.019; number of puffs = 12.5 (SD = 4.2) versus 17.0 (SD = 4.2), *t*_(7)_ =2.0, *P* = 0.084].

Regarding the comparison of Juul with the other EC products in product ratings, despite the small sample size, Juul received significantly more favourable ratings on several variables. It relieved urges to smoke faster than other ECs, was rated as more pleasant and was more likely to be recommended to friends (see Supporting information, Table S3).

## Discussion

When the US version of Juul and own cigarette are used *ad libitum* for 5 minutes after overnight abstinence, the nicotine delivery parameters of the two products are close to each other and different from other EC products. Compared to other EC products, Juul provides better nicotine delivery, better reduction of urges to smoke and better subjective ratings.

The findings are remarkable for several reasons. Until now, nicotine replacement devices struggled to match the PK profile of cigarettes. NRT products deliver less nicotine than cigarettes and do it more slowly. There is some evidence that faster‐acting products are more effective 24, but even the product with the most rapid nicotine delivery, nicotine nasal spray, delivers nicotine more slowly than cigarettes 25. One of the barriers to producing NRT with a better PK profile was local irritation, with stronger NRT products being less palatable 26. When ECs first appeared, their nicotine delivery was also low and slow 7. With improvements in battery power and atomizer construction the PK profile of EC devices is gradually improving 9, but the local irritation that hampered NRT development also seemed to apply here. Increasing nicotine content of EC aerosol typically increased the aversive effects of inhaling it. Against this background, the PK profile of Juul represents a substantial leap. A palatable device is now available that approaches cigarettes in nicotine delivery.

NRT has only limited therapeutic efficacy 24 and very low dependence potential 10. Regular use by non‐smokers is extremely rare. This was considered to be due to a lack of sensorimotor effects and lower and slower nicotine delivery compared to cigarettes. However, hypotheses were also generated linking the dependence potential of tobacco products to other chemicals in tobacco, such as monoamine oxidase inhibitors, that may reinforce nicotine effects or have reinforcing properties of their own 27, 28.

In this study, Juul reduced urges to smoke after overnight abstinence not significantly differently than cigarettes and faster than other EC products. This could suggest that the PK profile of nicotine delivery may be paramount. The conclusion, however, is only tentative. Smokers can react in a highly positive way to a smoking replacement experience that provides sufficient sensory input to generate a conditioned response, but if the central reward is incomplete, the effect dissipates over time 29. Juul may be approaching the central reward that smoking provides but it is also possible that, without other tobacco reinforcers, the effect weakens over time.

No data are currently available on the efficacy of Juul in smoking cessation. Our findings suggest that it is likely to be more effective than existing NRT and also more effective than older types of EC, but it remains to be seen whether its long‐term efficacy will be close to 20% or so that the best current medications achieve 30, or whether it will be in the much higher region of almost complete ‘smoking replacement’ 31.

The difference between the PK profile of Juul and of other types of EC devices was large, but this was not reflected in subjective ratings of nicotine delivery. It is noteworthy that when trying the product for the first time, experienced vapers thought that Juul delivered similar levels of nicotine as the other EC products. The nicotine salt formulation seems to blunt sensory clues to nicotine intake. Regarding central clues, we reported earlier that smokers do not detect nicotine content of products tried for the first time very well 19. As noted above, a repeated exposure seems necessary for smokers to dissociate conditioned and sensory effects from the central effects that are more important in driving the behaviour. Juul, however, obtained more favourable subjective ratings in other dimensions. The fact that Juul is outselling other EC products could be due partly to its early subjective superiority, but due more probably to smokers also gradually recognizing and appreciating its central effects.

The effects on smokers discussed so far can be seen as a positive development that is likely to generate better rates of quitting smoking. However, our findings also signal a potentially negative corollary: a higher attractiveness of the device to non‐smokers. As discussed in the Introduction, daily use of EC products among non‐smokers is very rare. No data are currently available on such use for Juul. There is a high degree of Juul experimentation among adolescents in the United States 3, and information should become available soon on the proportion of non‐smokers who try Juul and become daily users compared to the negligible proportion of non‐smokers who try other EC products and progress to daily use. If Juul shows a higher potential than other EC products to initiate regular nicotine use, careful analysis will be needed concerning the implications of such use on health, and of the balance between any detrimental effects of this and the positive effects of reduced smoking related mortality and morbidity generated by an increased rate of smoking cessation as well as the probable deflection of young people—who would otherwise become smokers—to Juul use.

Our study has several limitations. The sample was comprised mainly of males. Measures of subjective effects were limited to urge to smoke and product ratings. The sample size for the comparisons of Juul and other EC products was relatively small, although not unusually so for this type of study, and it was sufficient to detect a range of significant effects. At the time of the experiment, participants were mostly smoking only occasionally. If the experiment included light smokers, cigarette PK values could have been atypical, but the participants had a typical smoking history and their cigarette PK values correspond with those normally found in regular smokers 8. Some baseline nicotine levels suggested nicotine intake late at night or on the morning of the test, and this could have affected the results. In this ongoing project, where new products are tested when they appear, order of testing cannot be randomized and an order effect cannot be ruled out. Data were collected in dual users and the results may not generalize to non‐smokers who use Juul. The purpose of the study was to examine nicotine intake from *ad libitum* use, so the study does not provide information on nicotine intake per puff.

In summary, Juul is likely to be superior to previous EC products in helping smokers to quit smoking, but it may also have a higher potential to generate regular use in non‐smokers.

## Declaration of interests

P.H. has received research funding from and provided consultancy to Pfizer, a manufacturer of stop‐smoking medications. D.P. received research funding from Pfizer. The remaining authors have no conflicts of interest to declare.

EC = electronic cigarette; IQR = interquartile range.

## Supporting information


**Table S1** Cigarette brands used by participants.
**Table S2** Regression coefficient (b) and 95%CI from the mixed linear regression model exploring the effect of product (cigarette versus Juul), time and product*time on urges to smoke.
**Table S3** Ratings of Juul and other EC products (N = 8).
