# Supplementary material for: Nicotine delivery and users’ reactions to Juul compared with cigarettes and other e‐cigarette products
Source: Addiction. 2020 Jan 29;115(6):1141–8. doi: 10.1111/add.14936 (PMC7318270; doi:10.1111/add.14936)
Supplement: Supplementary file 1 — Table S1 Cigarette brands used by participants. Table S2 Regression coefficient (b) and 95%CI from the mixed linear regression model exploring the effect of product (cigarette versus Juul), time and product*time on urges to smoke. Table S3 Ratings of Juul and other EC products (N = 8). [file ADD-115-1141-s001.docx]

**Supplementary Files**

**Supplementary Table 1. Cigarette brands used by participants**

| **Cigarette Brand** | **N** | **Menthol** |
| --- | --- | --- |
| Amber Leaf | 1 | no |
| Benson & Hedges Blue | 1 | no |
| Golden Virginia Rolling Tobacco | 2 | no |
| Golden Virginia Smooth | 1 | no |
| Marlboro | 1 | no |
| Marlboro Ice blasts | 1 | yes |
| Marlboro Gold | 1 | no |
| Marlboro Red | 1 | no |
| Pall Mall | 1 | no |
| Pall Mall Blue | 2 | no |
| Pall Mall Red Capsule | 1 | yes |
| Rothman Blue | 1 | no |
| Rothman Silver | 1 | no |
| Rothmans Superking | 1 | no |
| Sovereign Dual | 1 | yes |
| Sterling Dual | 1 | yes |
| Winston Blue | 1 | no |
| Winston Classic | 1 | no |

**Supplementary Table 2.** **Regression coefficient (b) and 95%CI from the mixed linear regression model exploring the effect of product (cigarette vs. Juul), time and product*time on urges to smoke**

|  | **b (95% CI)** | **p** | **Wald test** |
| --- | --- | --- | --- |
| Time |  |  |  |
| 10 | 0.15 (-0.44 to 0.74) | 0.62 | Chi^2^(3) = 21.9,  p<0.001 |
| 15 | 0.65 (-0.04 to 1.34) | 0.06 |  |
| 30 | 1.75 (0.55 to 2.96) | 0.004 |  |
| Product |  |  |  |
| Juul | 0.20 (-0.94 to 1.33) | .73 | Chi^2^(1)=0.1,  p = 0.73 |
| Product*time |  |  |  |
| Juul @5 | 0.20 (-0.94 to 1.33) | 0.73 | Chi^2^(3)=2.8,  p=0.42 |
| Juul @10 | -0.10 (-1.14 to 0.94) | 0.85 |  |
| Juul @15 | 0.20 (-0.84 to 1.24) | 0.71 |  |
| Juul @30 | 0.40 (-0.96 to 1.76) | 0.57 |  |

**Supplementary Table 3: Ratings of Juul and other EC products (N=8)**

| **Product characteristic**  **Mean (SD)** | **Juul** | **Other EC** | **Difference *** | **Effect size **** |
| --- | --- | --- | --- | --- |
| Did it relieved your urge to smoke (1-10) | 9.1 (1.4) | 8.5 (1.2) | z=1.7, p=.09 | 0.60 |
| How quickly did any effect happen? (1-10) | 8.8 (1.0) | 7.7 (1.1) | z=-2.5  p=0.01 | 0.88 |
| Subjective nicotine delivery  (1=too little, 5=just right, 10= too much) | 6.3 (1.9) | 6.5 (1.2) | t(7)= -0.5,  p=.67 | 0.18 |
| Taste | 6.8 (2.4) | 5.0 (1.1) | t(7)=1.8, p =.11 | 0.64 |
| Pleasantness | 8.0 (2.1) | 5.7 (1.1) | t(7)=2.8, p=0.026 | 0.99 |
| Would recommend to friends | 8.4 (1.8) | 5.8 (1.0) | T(7)=4.3, p=0.003 | 1.52 |

** Paired t-test if parametric assumptions were met, Wilcoxon sign-ranked test if not*

*** Effect size was estimated using Cohen’s d test following paired t-test - t/sqrt(N) or r following a Wilcoxon signed rank test – z/sqrt(N).*
